# Cerebral blood flow in conscious rats and implications for therapeutic interventions

**DOI:** 10.1113/EP093381

**Published:** 2026-09-02

**Authors:** Tonja W. Emans, Courtney R. Brighouse, Jack T. Sloan, Fiona D. McBryde

**Affiliations:** ^1^ Manaaki Manawa – The Centre for Heart Research, Department of Physiology, Faculty of Medical and Health Sciences University of Auckland Auckland New Zealand

**Keywords:** cerebral blood flow, glymphatic function, hypertension

## Abstract

Cerebral blood flow (CBF) often becomes compromised in patients suffering from cardiovascular diseases, increasing the risk of neurodegenerative disease and vascular dementia. There is an urgent need for treatments to address cardiovascular risk without compromising brain perfusion, yet the impact of many existing treatments on CBF are not well understood. An exclusive focus on arterial blood pressure (BP) control against a background of cerebral vascular damage may miss an opportunity to restore cerebral haemodynamic regulation and mitigate risk to cognitive function. This Symposium Review focuses on the possibility and promise of assessing and optimising CBF during treatment for cardiovascular diseases. This is especially relevant given the new interest in pathways that protect brain health via cerebrospinal fluid exchange, such as the glymphatic system. Insights into the fundamental mechanisms connecting vascular motility and brain health can be gleaned from conscious, high‐fidelity measurements of CBF. We believe that CBF monitoring is crucial in the development of novel compounds, or re‐evaluation of current drugs.

## Abstract (highlights)

- **What is the topic of this review?**
  This review highlights the importance of the interactions between arterial pressure, cerebral blood flow, brain perfusion and cerebrospinal fluid movements, and the implications for selective and effective therapeutic interventions.
- **What advances does it highlight?**
  Conscious preclinical measurements of cerebral blood flow in combination with blood pressure hold promise to enable mechanistic insights into the progression of vascular damage and development of treatments to enhance brain health.

## 1 INTRODUCTION

Cerebral hypoperfusion commonly precedes neurodegeneration in models of cardiovascular disease (Tang et al., 2025; Wang et al., 2020) and is associated with cognitive decline and Alzheimer's risk in the general population (Wolters et al., 2017). Establishing causality is challenging, with some clinical studies suggest that blood pressure (BP) lowering treatments can compromise cerebral perfusion (Hart, 2016) while others show no change (van Rijssel et al., 2022). Thus, there is an urgent need to better understand the impact of treatments to address cardiovascular risk on brain perfusion. The impact of existing long‐term cardiovascular therapies on cerebral blood flow (CBF) is often not clearly defined because CBF assessment is challenging in both the clinical and preclinical setting, with the consequence that CBF measurements are often performed under anaesthesia in animal models. We measured the relationship between short‐term changes in arterial pressure (BP) and acute CBF in healthy awake rats and confirmed that CBF associates almost linearly with BP (Fong et al., 2021), which suggest that there is a risk of cerebral hypoperfusion during BP fluctuation, particularly during systemic hypertension, when long‐term CBF is already significantly decreased (Dijsselhof et al., 2025; Tamaki et al., 1995). CBF and cerebrospinal fluid (CSF) flow both rely on vascular motility and link between neurodegeneration and cardiovascular disease. This symposium review focuses on the importance of high‐fidelity assessment of CBF, a possible link with CSF clearance, and the possibility and promise of assessing and optimising CBF during treatments.

## 2 MEASURING CBF IN CONSCIOUS RATS

Measuring CBF is challenging due to the brain vasculature being ‘hidden’ inside the skull. Around 1880, the first brain blood pulsation measurements were made by Mosso, through an ‘accidentalmente’ opening in the skull of three people (Mosso, 1880). This was followed by relative measurements of CBF in dogs, cats and rabbits (Roy & Sherrington, 1890). Around 1940, the first quantifiable CBF measurements were performed in monkeys under anaesthesia, isolating internal carotid blood flow (Dumke & Schmidt, 1943). Later, Kety and Schmidt used the rate of nitrous gas absorption divided by the arterial–venous concentration difference over time to calculate CBF in animal models and conscious humans (Kety & Schmidt, 1948).

Based on these early studies, the concept of cerebral autoregulation emerged: that the cerebral vasculature will dilate or constrict in order to maintain a constant CBF during changes in BP (Lassen, 1959). The approaches used to create Lassen's ‘cerebral autoregulation’ curve are now regarded as reflecting ‘static’ autoregulation – the relationship between BP and CBF during a steady‐state (long‐term) level of BP, wherein CBF is relatively maintained around 10% of BP change (Claassen et al., 2021). The modern view differentiates between ‘static’ and ‘dynamic’ autoregulation. Dynamic autoregulation of CBF was discovered when techniques allowed for higher temporal resolution and describes the changes in CBF during rapidly changing BP (Brassard et al., 2021; Claassen et al., 2021). In order to understand the dynamic relationship between BP and CBF, techniques with beat‐to‐beat resolution are needed.

Anaesthetised CBF assessment in smaller animals, like rats, emerged in the 1970s (Hernandez et al., 1978; Matsumoto et al., 1975). Conscious CBF techniques today encompass laser Doppler flow (Barakat et al., 2024), magnetic resonance imaging (Sicard et al., 2003), positron emission tomography (Suzuki et al., 2021), SPECT imaging (Sugita et al., 2018), time‐transit flow probes (Fong et al., 2021), or indirectly infer CBF via measurements of intracranial pressure (Wittenberg et al., 2025). Conscious measurements of CBF revealed that most classes of anaesthesia substantially decrease CBF (Sicard et al., 2003; Suzuki et al., 2021) or change CBF sensitivity to changing BP (Hoffman et al., 1991).

**FIGURE 1:**
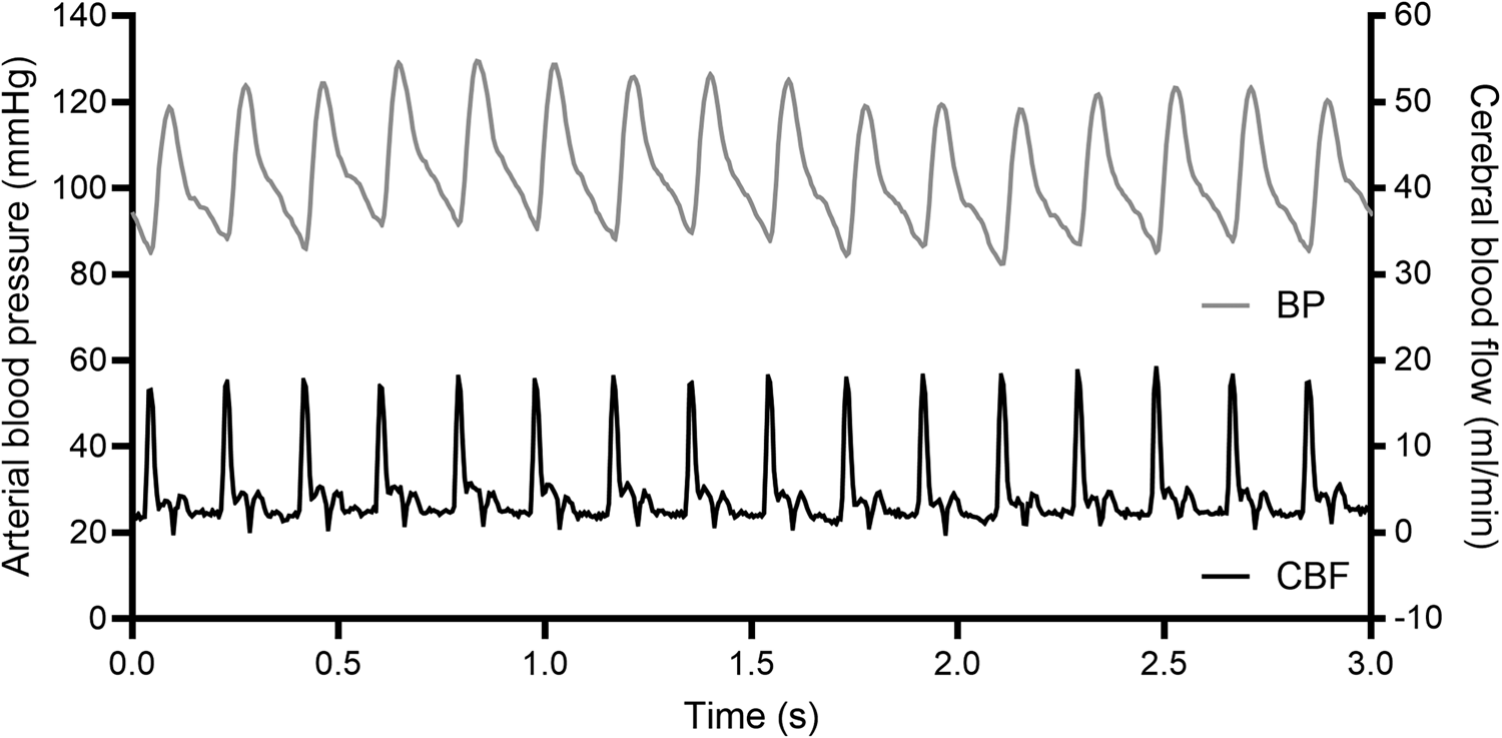
Typical volumetric cerebral blood flow waveform in a conscious Wistar rat. Measuring cerebral blood flow using perivascular flow probes chronically implanted around the carotid artery enables volumetric cerebral blood flow combined with arterial pressure. [Emans et al. unpublished.]

To capture the naturally oscillating nature of blood flow and the reciprocal interactions with arterial pressure, our lab has chosen to combine chronically implanted pressure telemeters with perivascular time‐transit flow probes in rats (Figure 1); a key advantage of this approach is the combination of high‐fidelity arterial pressure and volumetric blood flow in conscious, unrestrained rats (Emans et al., 2024; Fong et al., 2021).

During the ‘Arterial BP, Cerebral Perfusion and Brain Health’ symposium at the 40th congress of the International Union of Physiological Sciences in Frankfurt, we demonstrated the potential of this approach to reveal the complex dynamic relationships between BP and CBF. Given that a decline in global CBF and/or impaired dynamic regulation of the BP–CBF relationship frequently precedes neurodegeneration and cognitive impairment (Iadecola, 2013; Tang et al., 2025), our approach holds promise as an early read‐out of beneficial or detrimental effects of a given treatment, able to be applied in a range of preclinical disease models.

## 3 BRAIN PERFUSION AND CSF FLOW

Currently, a rapidly expanding area of research interest is the movement of CSF through the perivascular spaces to facilitate brain waste removal often referred to as ‘the glymphatic system’ (Iliff et al., 2012; Rennels et al., 1985). Studies have highlighted a tantalising putative link between glymphatic function and the development of neurodegenerative disorders (Y. R. Sun et al., 2025). Our knowledge of this system is almost solely drawn from experiments under anaesthesia, due to the need for invasive techniques and that glymphatic inflow occurs predominantly during sleep (Hauglund et al., 2025). The movement of CSF through the parenchyma clears toxic metabolic waste from the brain and appears to be dependent on arterial pulsation (Mestre et al., 2018). We and others infer that glymphatic inflow is likely to be influenced by CBF long term.

Recently, Hauglund *et al.* assessed the glymphatic function by combining blood and CSF tracer measurements without anaesthesia, using fibre photometry (Hauglund et al., 2025). They showed that waves of noradrenaline during NREM sleep induce slow oscillations of the cerebral vasculature that enhance the ‘pumping’ movements driving CSF through the perivascular space. CSF inflow has been found to be impaired in many vascular diseases, like systemic hypertension (C. Zhang et al., 2025; Mortensen et al., 2019; Xia et al., 2025), diabetes (Jiang et al., 2017) and heart failure (Kritsilis et al., 2025), vascular diseases which accompany reduced steady state CBF, and therefore the compliance of the global cerebral vasculature likely plays an important role in healthy brain clearance function.

Increased wall to lumen ratio in the main cerebral arteries leads to reduced CBF in systemic hypertension (Jordao et al., 2021) and disrupts the perivascular pump needed for CSF flow (Mestre et al., 2018). In our view, the common factor that directly links static CBF to CSF clearance, is the degree of vessel stiffness rather than level of acute CBF per se. This is supported by observations that isoflurane anaesthesia increases CBF but does not improve glymphatic clearance (Zhao et al., 2020). Similarly, acute vasodilation reduces CSF flow (Ge et al., 2026) and CSF clearance is most active during NREM sleep, which coincides with global CBF dipping at night‐time (Al‐Shama et al., 2024). The relationship between acute and chronic changes in BP, CBF and CSF flow remain incompletely understood, particularly when it comes to the impacts of therapeutic interventions.

## 4 THERAPEUTIC INTERVENTIONS TARGETING CBF

Therapeutic regimes to reduce cardiovascular risk, often target arterial BP and generally do not take CBF into consideration. Many anti‐hypertensive treatments will improve vascular motility, which is the shared connection between BP, CBF and CSF clearance. Despite the relationship of CBF to perfusion pressure, lowering BP by anti‐hypertensives does not appear to cause dangerous hypoperfusion (Christie et al., 2022; van Rijssel et al., 2022). In fact, intensive anti‐hypertensive treatment targeting systolic pressure <120 mmHg can increase CBF over time (Dolui et al., 2022) and reduce cognitive decline (den Brok et al., 2021). However, there is evidence showing differential effects on CBF with different classes of drugs. In hypertensive rats, BP was controlled similarly by calcium channel blockade (amlodipine) versus beta‐blockade (atenolol) long term; however, cerebral vascular resistance was reduced by amlodipine only (Naessens et al., 2023). Inhibition of inflammation through the TLR4/NF‐κB pathway in a pre‐clinical model of ischaemic stroke increases CBF (Zhang et al., 2023) and reduces neuronal loss but does not reduce BP (J. Sun et al., 2025). During brain renin–angiotensin system (RAS) activation, hydralazine normalizes BP but does not improve cognitive function (Wu et al., 2025). Taken together, these results suggest that BP control offers neuroprotection only when CBF is improved or maintained.

Angiotensin‐converting enzyme inhibitors have been reported to shift CBF autoregulation to lower pressures, improving cerebral perfusion (Waldemar & Paulson, 1989). Better rehabilitation outcomes in hypertensive stroke patients during angiotensin II receptor blockade were associated with measurable increases in CBF rather than changes in BP per se (Matsumoto et al., 2009). RAS drugs that can cross the blood–brain barrier (BBB) offer more cognitive protection than equivalent non‐BBB‐crossing RAS drugs (Ho et al., 2021). Therefore, targeting the brain specific RAS has been proposed as a novel treatment for Alzheimer's disease (Loera‐Valencia et al., 2021). Targeting brain RAS improved CSF clearance and CBF in a model of traumatic brain injury (X. Zhang et al., 2025), wherein global CBF was a better indicator for brain damage than BP. One possibility is that BBB‐penetrative RAS treatments could work in part by improving glymphatic function.

Physical exercise has been shown to improve cognitive health in a broad range of populations (Singh et al., 2025). This might be due to increases in CBF. In hypertensive rats, exercise improves global CBF by increasing capillary density in the brain, decreasing cerebral vascular resistance and normalising systemic hypertension induced vascular rarefaction (Jordao et al., 2021). To protect the brain from cognitive decline, CBF needs to be maintained in brain areas responsible for cognitive function, like the hippocampus and the prefrontal cortex. ‘Bypassing’ full exercise by electrical stimulation of muscular mass only increases regional CBF in the areas related to muscular control but not in the cognitive areas (Chaney et al., 2022). Therefore, it seems that exercise warrants cognitive engagement for its benefits. Likewise, cognitive training, without physical element, leads to increases in resting CBF and improvement of focus in older adults (Mozolic et al., 2010).

The ability of the cerebral vasculature to conduct physiological oscillations appears fundamental to support CBF and glymphatic clearance, reinforcing the tight interactions between vascular health, brain waste removal and neuronal function. While anti‑hypertensive therapies are generally recognized for preserving CBF in hypertensive patients, the potential benefits extend beyond BP control. We believe that a solid understanding of CBF haemodynamic control is crucial in the search for validating and repurposing current drugs and treatments with the specific goal of concurrently optimising cerebral perfusion and brain health.

## AUTHOR CONTRIBUTIONS

All authors contributed to the conception, design, and draft of the work and revising it critically for important intellectual content. All authors have approved this review in its final form and agree to be accountable for all aspects of the work. All persons designated as authors qualify for authorship, and all those who qualify for authorship are listed.

## CONFLICT OF INTEREST

All authors have no conflicts to declare.

## GENERATIVE AI STATEMENT

The authors confirm that no artificial intelligence tools, including large language models (LLMs), were used in the drafting or revision of this manuscript. All content was conceived, written, and approved solely by the authors.

## References

[eph70457-bib-0001] Al‐Shama, R. F. M. , Uleman, J. F. , Pereira, M. , Claassen, J. , & Dresler, M. (2024). Cerebral blood flow in sleep: A systematic review and meta‐analysis. Sleep Medicine Reviews, 77, 101977. 39096646 10.1016/j.smrv.2024.101977

[eph70457-bib-0002] Barakat, R. M. , Turcani, M. , Al‐Khaledi, G. , Kilarkaje, N. , Al‐Sarraf, H. , Sayed, Z. , & Redzic, Z. (2024). Low oxygen in inspired air causes severe cerebrocortical hypoxia and cell death in the cerebral cortex of awake rats. Neuroscience Letters, 818, 137515. 37865187 10.1016/j.neulet.2023.137515

[eph70457-bib-0003] Brassard, P. , Labrecque, L. , Smirl, J. D. , Tymko, M. M. , Caldwell, H. G. , Hoiland, R. L. , Lucas, S. J. E. , Denault, A. Y. , Couture, E. J. , & Ainslie, P. N. (2021). Losing the dogmatic view of cerebral autoregulation. Physiological Reports, 9(15), e14982. 34323023 10.14814/phy2.14982 PMC8319534

[eph70457-bib-0004] Chaney, R. , Garnier, P. , Quirie, A. , Martin, A. , Prigent‐Tessier, A. , & Marie, C. (2022). Region‐dependent increase of cerebral blood flow during electrically induced contraction of the hindlimbs in rats. Frontiers in Physiology, 13, 811118. 35492591 10.3389/fphys.2022.811118 PMC9040888

[eph70457-bib-0005] Christie, I. N. , Windsor, R. , Mutsaerts, H. J. , Tillin, T. , Sudre, C. H. , Hughes, A. D. , Golay, X. , Gourine, A. V. , & Hosford, P. S. (2022). Cerebral perfusion in untreated, controlled, and uncontrolled hypertension. Journal of Cerebral Blood Flow and Metabolism, 42(12), 2188–2190. 36113055 10.1177/0271678X221124644 PMC7613835

[eph70457-bib-0006] Claassen, J. , Thijssen, D. H. J. , Panerai, R. B. , & Faraci, F. M. (2021). Regulation of cerebral blood flow in humans: Physiology and clinical implications of autoregulation. Physiological Reviews, 101(4), 1487–1559. 33769101 10.1152/physrev.00022.2020 PMC8576366

[eph70457-bib-0007] den Brok, M. , van Dalen, J. W. , Abdulrahman, H. , Larson, E. B. , van Middelaar, T. , van Gool, W. A. , Moll van Charante, E. P. , & Richard, E. (2021). Antihypertensive medication classes and the risk of dementia: A systematic review and network meta‐analysis. Journal of the American Medical Directors Association, 22(7), 1386–1395. e1315. 33460618 10.1016/j.jamda.2020.12.019

[eph70457-bib-0008] Dijsselhof, M. B. , Holtrop, J. , James, S. N. , Sudre, C. H. , Lu, K. , Lorenzini, L. , Collij, L. E. , Scott, C. J. , Manning, E. N. , Thomas, D. L. , Richards, M. , Hughes, A. D. , Cash, D. M. , Barkhof, F. , Schott, J. M. , Petr, J. , & Mutsaerts, H. J. (2025). Associations of life‐course cardiovascular risk factors with late‐life cerebral hemodynamics. Journal of Cerebral Blood Flow and Metabolism, 45(4), 765–778. 39552078 10.1177/0271678X241301261 PMC11571377

[eph70457-bib-0009] Dolui, S. , Detre, J. A. , Gaussoin, S. A. , Herrick, J. S. , Wang, D. J. J. , Tamura, M. K. , Cho, M. E. , Haley, W. E. , Launer, L. J. , Punzi, H. A. , Rastogi, A. , Still, C. H. , Weiner, D. E. , Wright, J. T., Jr. , Williamson, J. D. , Wright, C. B. , Bryan, R. N. , Bress, A. P. , Pajewski, N. M. , & Nasrallah, I. M. (2022). Association of intensive vs standard blood pressure control with cerebral blood flow: Secondary analysis of the SPRINT MIND randomized clinical trial. JAMA Neurology, 79(4), 380–389. 35254390 10.1001/jamaneurol.2022.0074 PMC8902686

[eph70457-bib-0010] Dumke, P. , & Schmidt, C. (1943). Quantitative measurements of cerebral blood flow in the macaque monkey. American Journal of Physiology, 138, 421–431.

[eph70457-bib-0011] Emans, T. W. , Moraes, D. J. A. , Ben‐Tal, A. , Barrett, C. J. , Paton, J. F. R. , & McBryde, F. D. (2024). Forgotten circulation: Reduced mesenteric venous capacitance in hypertensive rats is improved by decreasing sympathetic activity. Hypertension, 81(4), 823–835. 38380519 10.1161/HYPERTENSIONAHA.123.21878

[eph70457-bib-0012] Fong, D. , Gradon, K. , Barrett, C. J. , Guild, S. J. , Tzeng, Y. C. , Paton, J. F. R. , & McBryde, F. D. (2021). A method to evaluate dynamic cerebral pressure‐flow relationships in the conscious rat. Journal of Applied Physiology, 131(4), 1361–1369. 34498945 10.1152/japplphysiol.00289.2021

[eph70457-bib-0013] Ge, H. , Gu, X. , Wang, Z. , Tan, S. , Jiao, B. , Zhang, L. , Yang, Y. , Li, W. , Xie, J. , & Bai, R. (2026). Anesthetics modulate cerebrospinal fluid efflux pathways in mice by altering perineural and perivascular spaces. NMR in Biomedicine, 39(2), e70222. 41469234 10.1002/nbm.70222

[eph70457-bib-0014] Hart, E. C. (2016). Human hypertension, sympathetic activity and the selfish brain. Experimental Physiology, 101(12), 1451–1462. 27519960 10.1113/EP085775

[eph70457-bib-0015] Hauglund, N. L. , Andersen, M. , Tokarska, K. , Radovanovic, T. , Kjaerby, C. , Sorensen, F. L. , Bojarowska, Z. , Untiet, V. , Ballestero, S. B. , Kolmos, M. G. , Weikop, P. , Hirase, H. , & Nedergaard, M. (2025). Norepinephrine‐mediated slow vasomotion drives glymphatic clearance during sleep. Cell, 188(3), 606–622. e617. 39788123 10.1016/j.cell.2024.11.027 PMC12340670

[eph70457-bib-0016] Hernandez, M. J. , Brennan, R. W. , & Bowman, G. S. (1978). Cerebral blood flow autoregulation in the rat. Stroke; A Journal of Cerebral Circulation, 9(2), 150–154. 10.1161/01.str.9.2.150 644608

[eph70457-bib-0017] Ho, J. K. , Moriarty, F. , Manly, J. J. , Larson, E. B. , Evans, D. A. , Rajan, K. B. , Hudak, E. M. , Hassan, L. , Liu, E. , Sato, N. , Hasebe, N. , Laurin, D. , Carmichael, P. H. , & Nation, D. A. (2021). Blood‐brain barrier crossing renin‐angiotensin drugs and cognition in the elderly: A meta‐analysis. Hypertension, 78(3), 629–643. 34148364 10.1161/HYPERTENSIONAHA.121.17049 PMC9009861

[eph70457-bib-0018] Hoffman, W. E. , Edelman, G. , Kochs, E. , Werner, C. , Segil, L. , & Albrecht, R. F. (1991). Cerebral autoregulation in awake versus isoflurane‐anesthetized rats. Anesthesia and Analgesia, 73(6), 753–757. 1952176 10.1213/00000539-199112000-00013

[eph70457-bib-0019] Iadecola, C. (2013). The pathobiology of vascular dementia. Neuron, 80(4), 844–866. 24267647 10.1016/j.neuron.2013.10.008 PMC3842016

[eph70457-bib-0020] Iliff, J. J. , Wang, M. , Liao, Y. , Plogg, B. A. , Peng, W. , Gundersen, G. A. , Benveniste, H. , Vates, G. E. , Deane, R. , Goldman, S. A. , Nagelhus, E. A. , & Nedergaard, M. (2012). A paravascular pathway facilitates CSF flow through the brain parenchyma and the clearance of interstitial solutes, including amyloid beta. Science Translational Medicine, 4(147), 147ra111. 10.1126/scitranslmed.3003748 PMC3551275 22896675

[eph70457-bib-0021] Jiang, Q. , Zhang, L. , Ding, G. , Davoodi‐Bojd, E. , Li, Q. , Li, L. , Sadry, N. , Nedergaard, M. , Chopp, M. , & Zhang, Z. (2017). Impairment of the glymphatic system after diabetes. Journal of Cerebral Blood Flow and Metabolism, 37(4), 1326–1337. 27306755 10.1177/0271678X16654702 PMC5453454

[eph70457-bib-0022] Jordao, M. T. , Ceroni, A. , & Michelini, L. C. (2021). Perfusion of brain preautonomic areas in hypertension: Compensatory absence of capillary rarefaction and protective effects of exercise training. Frontiers in Physiology, 12, 773415. 34975525 10.3389/fphys.2021.773415 PMC8716837

[eph70457-bib-0023] Kety, S. S. , & Schmidt, C. F. (1948). The nitrous oxide method for the quantitative determination of cerebral blood flow in man: Theory, procedure and normal values. Journal of Clinical Investigation, 27(4), 476–483. 16695568 10.1172/JCI101994 PMC439518

[eph70457-bib-0024] Kritsilis, M. , Vanherle, L. , Rosenholm, M. , In ’t Zandt, R. , Yao, Y. , Swanberg, K. M. , Weikop, P. , Gottschalk, M. , Shanbhag, N. C. , Luo, J. , Boster, K. , Nedergaard, M. , Meissner, A. , & Lundgaard, I. (2025). Loss of glymphatic homeostasis in heart failure. Brain, 148(3), 985–1000. 39693238 10.1093/brain/awae411 PMC11884761

[eph70457-bib-0025] Lassen, N. A. (1959). Cerebral blood flow and oxygen consumption in man. Physiological Reviews, 39(2), 183–238. 13645234 10.1152/physrev.1959.39.2.183

[eph70457-bib-0026] Loera‐Valencia, R. , Eroli, F. , Garcia‐Ptacek, S. , & Maioli, S. (2021). Brain renin‐angiotensin system as novel and potential therapeutic target for Alzheimer's disease. International Journal of Molecular Sciences, 22(18), 10139. 34576302 10.3390/ijms221810139 PMC8468637

[eph70457-bib-0027] Matsumoto, A. , Namon, R. , Utsunomiya, Y. , Kogure, K. , Scheinberg, P. , & Reinmuth, O. M. (1975). The measurement of cerebral blood flow in the rat. Stroke, 6(6), 630–637. 128160 10.1161/01.str.6.6.630

[eph70457-bib-0028] Matsumoto, S. , Shimodozono, M. , Miyata, R. , & Kawahira, K. (2009). Benefits of the angiotensin II receptor antagonist olmesartan in controlling hypertension and cerebral hemodynamics after stroke. Hypertension Research, 32(11), 1015–1021. 19745828 10.1038/hr.2009.143

[eph70457-bib-0029] Mestre, H. , Tithof, J. , Du, T. , Song, W. , Peng, W. , Sweeney, A. M. , Olveda, G. , Thomas, J. H. , Nedergaard, M. , & Kelley, D. H. (2018). Flow of cerebrospinal fluid is driven by arterial pulsations and is reduced in hypertension. Nature Communications, 9(1), 4878. 10.1038/s41467-018-07318-3 PMC6242982 30451853

[eph70457-bib-0030] Mortensen, K. N. , Sanggaard, S. , Mestre, H. , Lee, H. , Kostrikov, S. , Xavier, A. L. R. , Gjedde, A. , Benveniste, H. , & Nedergaard, M. (2019). Impaired glymphatic transport in spontaneously hypertensive rats. Journal of Neuroscience, 39(32), 6365–6377. 31209176 10.1523/JNEUROSCI.1974-18.2019 PMC6687896

[eph70457-bib-0031] Mosso, A. (1880). Sulla circolazione del sangue nel cervello dell'uomo. Atti della Reale Accademia dei Lincei. Memorie della Classe di Scienze Fisiche, Matematiche e Naturali, 3, 237–358.

[eph70457-bib-0032] Mozolic, J. L. , Hayasaka, S. , & Laurienti, P. J. (2010). A cognitive training intervention increases resting cerebral blood flow in healthy older adults. Frontiers in Human Neuroscience, 4, 16. 20300200 10.3389/neuro.09.016.2010 PMC2841485

[eph70457-bib-0033] Naessens, D. M. P. , de Vos, J. , Richard, E. , Wilhelmus, M. M. M. , Jongenelen, C. A. M. , Scholl, E. R. , van der Wel, N. N. , Heijst, J. A. , Teunissen, C. E. , Strijkers, G. J. , Coolen, B. F. , VanBavel, E. , & Bakker, E. (2023). Effect of long‐term antihypertensive treatment on cerebrovascular structure and function in hypertensive rats. Scientific Reports, 13(1), 3481. 36859481 10.1038/s41598-023-30515-0 PMC9977931

[eph70457-bib-0034] Rennels, M. L. , Gregory, T. F. , Blaumanis, O. R. , Fujimoto, K. , & Grady, P. A. (1985). Evidence for a ‘paravascular’ fluid circulation in the mammalian central nervous system, provided by the rapid distribution of tracer protein throughout the brain from the subarachnoid space. Brain Research, 326(1), 47–63. 3971148 10.1016/0006-8993(85)91383-6

[eph70457-bib-0035] Roy, C. S. , & Sherrington, C. S. (1890). On the Regulation of the Blood‐supply of the Brain. The Journal of Physiology, 11(1–2), 85–158. 117. 10.1113/jphysiol.1890.sp000321 PMC1514242 16991945

[eph70457-bib-0036] Sicard, K. , Shen, Q. , Brevard, M. E. , Sullivan, R. , Ferris, C. F. , King, J. A. , & Duong, T. Q. (2003). Regional cerebral blood flow and BOLD responses in conscious and anesthetized rats under basal and hypercapnic conditions: Implications for functional MRI studies. Journal of Cerebral Blood Flow and Metabolism, 23(4), 472–481. 12679724 10.1097/01.WCB.0000054755.93668.20 PMC2989608

[eph70457-bib-0037] Singh, B. , Bennett, H. , Miatke, A. , Dumuid, D. , Curtis, R. , Ferguson, T. , Brinsley, J. , Szeto, K. , Petersen, J. M. , Gough, C. , Eglitis, E. , Simpson, C. E. , Ekegren, C. L. , Smith, A. E. , Erickson, K. I. , & Maher, C. (2025). Effectiveness of exercise for improving cognition, memory and executive function: A systematic umbrella review and meta‐meta‐analysis. British Journal of Sports Medicine, 59(12), 866–876. 40049759 10.1136/bjsports-2024-108589 PMC12229068

[eph70457-bib-0038] Sugita, T. , Kondo, Y. , Ishino, S. , Mori, I. , Horiguchi, T. , Ogawa, M. , & Magata, Y. (2018). Evaluation of drug effects on cerebral blood flow and glucose uptake in un‐anesthetized and un‐stimulated rats: Application of free‐moving apparatus enabling to keep rats free during PET/SPECT tracer injection and uptake. Nuclear Medicine Communications, 39(8), 753–760. 29771718 10.1097/MNM.0000000000000863 PMC6075887

[eph70457-bib-0039] Sun, J. , Su, Z. , Ji, Y. , Wang, F. , Zhao, J. , Zhang, J. , & Li, L. (2025). DIA‐based proteomics reveals anti‐inflammatory role of DL‐3‐n‐butylphthalide in cerebral small vessel disease‐induced brain injury in hypertensive rat. Translational Neuroscience, 16(1), 20250381. 40919222 10.1515/tnsci-2025-0381 PMC12413627

[eph70457-bib-0040] Sun, Y. R. , Lv, Q. K. , Liu, J. Y. , Wang, F. , & Liu, C. F. (2025). New perspectives on the glymphatic system and the relationship between glymphatic system and neurodegenerative diseases. Neurobiology of Disease, 205, 106791. 39778750 10.1016/j.nbd.2025.106791

[eph70457-bib-0041] Suzuki, C. , Kosugi, M. , & Magata, Y. (2021). Conscious rat PET imaging with soft immobilization for quantitation of brain functions: Comprehensive assessment of anesthesia effects on cerebral blood flow and metabolism. European Journal of Nuclear Medicine and Molecular Imaging Research, 11(1), 46. 10.1186/s13550-021-00787-6 PMC8106566 33963948

[eph70457-bib-0042] Tamaki, K. , Nakai, M. , Yokota, T. , & Ogata, J. (1995). Effects of aging and chronic hypertension on cerebral blood flow and cerebrovascular CO_2_ reactivity in the rat. Gerontology, 41(1), 11–17. 7737529 10.1159/000213657

[eph70457-bib-0043] Tang, C. , Border, J. J. , Zhang, H. , Gregory, A. , Bai, S. , Fang, X. , Liu, Y. , Wang, S. , Hwang, S. H. , Gao, W. , Morgan, G. C. , Smith, J. , Bunn, D. , Cantwell, C. , Wagner, K. M. , Morisseau, C. , Yang, J. , Shin, S. M. , O'Herron, P. , … Fan, F. (2025). Inhibition of soluble epoxide hydrolase ameliorates cerebral blood flow autoregulation and cognition in Alzheimer's disease and diabetes‐related dementia rat models. GeroScience, 47(3), 4429–4449. 39903369 10.1007/s11357-025-01550-8 PMC12181608

[eph70457-bib-0044] van Rijssel, A. E. , Stins, B. C. , Beishon, L. C. , Sanders, M. L. , Quinn, T. J. , Claassen, J. , & de Heus, R. A. A. (2022). Effect of antihypertensive treatment on cerebral blood flow in older adults: A systematic review and meta‐analysis. Hypertension, 79(5), 1067–1078. 35193363 10.1161/HYPERTENSIONAHA.121.18255 PMC8997667

[eph70457-bib-0045] Waldemar, G. , & Paulson, O. B. (1989). Angiotensin converting enzyme inhibition and cerebral circulation–a review. British Journal of Clinical Pharmacology, 28(2), 177S–182S. 2690908 10.1111/j.1365-2125.1989.tb03593.x PMC1379863

[eph70457-bib-0046] Wang, S. , Lv, W. , Zhang, H. , Liu, Y. , Li, L. , Jefferson, J. R. , Guo, Y. , Li, M. , Gao, W. , Fang, X. , Paul, I. A. , Rajkowska, G. , Shaffery, J. P. , Mosley, T. H. , Hu, X. , Liu, R. , Wang, Y. , Yu, H. , Roman, R. J. , & Fan, F. (2020). Aging exacerbates impairments of cerebral blood flow autoregulation and cognition in diabetic rats. GeroScience, 42(5), 1387–1410. 32696219 10.1007/s11357-020-00233-w PMC7525432

[eph70457-bib-0047] Wittenberg, P. , McBryde, F. D. , Korsak, A. , Rodrigues, K. L. , Paton, J. F. R. , Marina, N. , & Gourine, A. V. (2025). On the regulation of arterial blood pressure by an intracranial baroreceptor mechanism. The Journal of Physiology, 603(9), 2517–2532. 39924875 10.1113/JP285082 PMC7617620

[eph70457-bib-0048] Wolters, F. J. , Zonneveld, H. I. , Hofman, A. , van der Lugt, A. , Koudstaal, P. J. , Vernooij, M. W. , & Ikram, M. A. , Heart‐Brain Connection Collaborative Research Group . (2017). Cerebral perfusion and the risk of dementia: A population‐based study. Circulation, 136(8), 719–728. 28588075 10.1161/CIRCULATIONAHA.117.027448

[eph70457-bib-0049] Wu, H. , Qiu, Z. , Mu, J. , Wang, Y. , Wang, J. , Han, Y. , Yang, R. , Yuan, S. , Yuan, M. , Yang, R. , Chen, X. , Sun, Q. , Li, F. , Xiao, L. , Zhang, M. , & Xu, J. (2025). Salt‐sensitive hypertension promotes neuronal mitochondrial stress and neurodegenerative alterations via neuro‐vascular metabolic reprogramming and local RAS signaling. Journal of Neuroinflammation, 22(1), 220. 41039568 10.1186/s12974-025-03533-0 PMC12490161

[eph70457-bib-0050] Xia, Y. , Lyu, C. , Chen, P. , Jiang, Y. , Qu, C. , Lyu, X. , Cheng, Z. , & Gao, B. (2025). The glymphatic system was impaired in spontaneously hypertensive rats. Scientific Reports, 15(1), 18321. 40419570 10.1038/s41598-025-02054-3 PMC12106702

[eph70457-bib-0051] Zhang, C. , Song, C. , Sheng, S. , Pan, L. , Sun, L. , & Xing, W. (2025). Reduced DTI‐ALPS in H‐type hypertension: Insights into perivascular space function. Frontiers in Neurology, 16, 1536001. 40255895 10.3389/fneur.2025.1536001 PMC12006006

[eph70457-bib-0052] Zhang, H. , Wang, L. , Zhu, B. , Yang, Y. , Cai, C. , Wang, X. , Deng, L. , He, B. , Cui, Y. , & Zhou, W. (2023). A comparative study of the neuroprotective effects of dl‐3‐n‐butylphthalide and edaravone dexborneol on cerebral ischemic stroke rats. European Journal of Pharmacology, 951, 175801. 37207969 10.1016/j.ejphar.2023.175801

[eph70457-bib-0053] Zhang, X. , Sun, B. , Li, W. , Liu, T. , Li, W. , Chen, B. , He, C. , Liu, Q. , Zhu, S. , & Wang, H. (2025). Enhancing glymphatic transport through angiotensin II type 2 receptor activation promotes neurological recovery after traumatic brain injury. Theranostics, 15(18), 9775–9792. 41041052 10.7150/thno.117743 PMC12486419

[eph70457-bib-0054] Zhao, G. , Han, H. , Yang, J. , Sun, M. , Cui, D. , Li, Y. , Gao, Y. , & Zou, J. (2020). Brain interstitial fluid drainage and extracellular space affected by inhalational isoflurane: In comparison with intravenous sedative dexmedetomidine and pentobarbital sodium. Science China Life Sciences, 63(9), 1363–1379. 32133594 10.1007/s11427-019-1633-3

